# Sub-Nanosecond Single Mode-Locking Pulse Generation in an Idler-Resonant Intracavity KTA Optical Parametric Oscillator Driven by a Dual-Loss-Modulated Q-Switched and Mode-Locked Laser with an Acousto-Optic Modulator and MoWS_2_

**DOI:** 10.3390/nano14181491

**Published:** 2024-09-13

**Authors:** Chao Han, Hongwei Chu, Tianli Feng, Shengzhi Zhao, Dechun Li, Han Zhang, Jia Zhao, Weiping Huang

**Affiliations:** School of Information Science and Engineering, Shandong University, Qingdao 266237, China; hanchao370@mail.sdu.edu.cn (C.H.); hongwei.chu@sdu.edu.cn (H.C.); tlfeng@sdu.edu.cn (T.F.); dechun@sdu.edu.cn (D.L.); zhaojia@sdu.edu.cn (J.Z.); wphuang_canada@hotmail.com (W.H.)

**Keywords:** MoWS_2_ saturable absorber, optical parametric oscillator, rate equations, Q-switched and mode-locked, single mode-locking pulse

## Abstract

The synthesis of 2D MoWS_2_ nanosheets involved the liquid-phase exfoliation technique was explored in this paper. The nonlinear optical response of MoWS_2_ was characterized in the 1 µm wavelength range, and its suitability as a saturable absorber (SA) was confirmed. Experimental demonstrations were conducted by using MoWS_2_ as an SA in an idler-resonant intracavity KTA optical parametric oscillator (OPO) driven by a dual-loss-modulated Q-switched and mode-locked (QML) YVO_4_/Nd:YVO_4_ laser with an acousto-optic modulator (AOM). By appropriately tuning the pump power and the AOM repetition rate, the Q-switched envelope pulse widths for the signal and idler waves could be significantly reduced to be shorter than the cavity round-trip transit time, i.e., the interval between two neighboring mode-locking pulses. Consequently, this enabled the generation of sub-nanosecond single mode-locking pulses with a low repetition rate, high pulse energy, and remarkable stability. With a repetition rate of 1 kHz and maximal pulse energies of 318 µJ and 169 µJ, respectively, sub-nanosecond single mode-locking pulses of the signal and idler waves were generated. The theoretical model was established using coupled rate equations with a Gaussian spatial distribution approximation. The numerical simulation results for generating sub-nanosecond single mode-locking pulses for the signal and idler waves within their respective Q-switched envelopes aligned fundamentally with the experimental results, proving that MoWS_2_ can be a potential nanomaterial for further optoelectronic applications.

## 1. Introduction

Sub-nanosecond eye-safe near-infrared (1.5 µm) and mid-infrared (3–5 µm) lasers with high pulse energy, stability, and a low repetition rate offer significant advantages for various practical applications, including atmospheric monitoring, remote sensing, and optoelectronic countermeasures [1,2]. The use of nonlinear optical (NLO) crystals allows for the effective generation of near-infrared and mid-infrared lasers through nonlinear frequency conversion. Among these methods, the optical parametric oscillator (OPO) stands out for its compact, simple, and robust configuration, making it a widely used tool in infrared laser applications. The continuous-wave mode-locking (CWML) technique is commonly applied to generate ultrashort pulses. However, in CWML lasers, the energy for each pulse is relatively low due to the high repetition rate. For CWML lasers, the typical pulse energy varies from pJ to tens of nJ, whereas the repetition rate is typically restricted by the cavity length and measured in tens of MHz. Additionally, the high repetition rate can lead to significant thermal lensing, resulting in heat accumulation in materials during optical nonlinearity measurements. To address this, efforts have been made to develop single passively Q-switched and mode-locked (QML) lasers. These QML lasers operate with a repetition rate primarily determined by saturable absorption. However, achieving a modulation depth close to 100% with these lasers remains a challenge, and maintaining amplitude stability beneath the Q-switched envelope is an ongoing concern.

The integration of an active modulator into a passively QML laser, termed dual-loss modulation [3,4], allows control over the envelope’s repetition rate. In dual-loss-modulated QML lasers, the Q-switched envelope’s repetition rate is regulated by the active modulator, while the mode-locking (ML) pulses depend on both the active modulation and the saturable absorption, leading to a 100% modulation depth. However, given the presence of multiple ML pulses within a Q-switched envelope, the average peak power for these ML pulses tends to be relatively low, typically in the range of 0.1 to tens of kW. This limitation has prompted efforts to achieve higher peak power ML lasers by reducing the number of ML pulses within a Q-switched envelope. The dual-loss-modulated QML laser, distinguished by its high peak power, sub-nanosecond pulse duration, and precise repetition rate adjustment, has emerged as an exceptional pump source for intracavity OPOs (IOPOs). Consequently, an IOPO pumped by a dual-loss-modulated QML laser has been proposed, with both theoretical simulations and experimental results demonstrating the generation of a signal beam characterized by a narrow pulse width and high average power [5].

In recent years, a variety of one-dimensional and two-dimensional (2D) nanomaterials have emerged and developed as potential candidates for OPO systems driven by Nd-doped lasers. These materials include carbon nanotubes [6], graphene [7], and transition metal dichalcogenides (TMDs) [8]. Among the TMDs, novel alloyed TMD species have gained significant interest due to their thermodynamic reliability, intriguing electrical characteristics, and layer-dependent properties [9,10,11]. These alloyed TMDs offer extensive possibilities for various applications such as field-effect transistors [12], hydrogen evolution catalysts [13], and optoelectronic devices [14]. For instance, it has been demonstrated that the band gap of 2D Mo_(1−x)_W_x_S_2_ monolayer alloys can be adjusted from 1.82 eV (x = 0.2) to 1.99 eV (x = 1) by altering the elemental composition [15]. Such enhanced freedom introduced by alloying components might significantly improve the performance of ultrashort laser pulse generation. Mo_(1−x)_W_x_S_2_ alloys have been theoretically and experimentally proven to possess broadband saturable absorption characteristics, serving as promising saturable absorbers (SAs) [16,17,18,19]. Yan et al. reported a passively Q-switched Er:YAG laser employing multilayer Mo_0.5_W_0.5_S_2_ (MoWS_2_), generating microsecond pulses at 1645 nm [16]. Ahmad et al. demonstrated passively Q-switched fiber lasers at various wavelengths using Mo_0.8_W_0.2_S_2_ thin films [17,18]. Niu et al. presented a 2 μm doubly Q-switched mode-locked (DQML) Tm:YAP laser incorporating an electro-optic modulator and Mo_0.5_W_0.5_S_2_ SA, achieving a pulse width of 205 ns and a peak power of 2.44 kW [19]. However, to the best of our knowledge, there has been no associated experimental study or accompanying theoretical analysis on OPOs driven by QML Nd-doped solid-state lasers based on ternary Mo_(1−x)_W_x_S_2_ alloys.

In this paper, 2D MoWS_2_ nanosheets were synthesized through the liquid-phase exfoliation method, followed by an evaluation of their nonlinear optical response. An LD-pumped idler-resonant KTA-based IOPO driven by a dual-loss-modulated QML laser with an acousto-optic modulator (AOM) and MoWS_2_ SA was constructed within this research framework. Under a certain pump power, sub-nanosecond single mode-locking pulses of the signal and idler waves were obtained, respectively. Leveraging the QML pulses’ fluctuation mechanism and assuming a Gaussian spatial distribution, a set of coupled rate equations governing the diode-pumped idler-resonant IOPO, driven by a dual-loss-modulated QML laser with AOM and MoWS_2_ SA, were derived. Our numerical simulation results are fundamentally in agreement with the experimental values. These investigations underscore the promising potential of MoWS_2_ as a highly effective nanomaterial for advanced ultrafast solid-state laser systems.

## 2. Materials and Methods

### 2.1. Fabrication and Characterizations of 2D MoWS_2_ SA

A liquid-phase exfoliation process was employed to produce MoWS_2_ nanosheets [20]. Initially, 10 mg of synthesized MoWS_2_ single crystals (synthesized by chemical-vapor transport technique [21]) were dispersed in 10 mL of isopropyl alcohol. Ultrasonic vibration was employed for 6 h to facilitate the exfoliation of nanosheets, followed by centrifugal separation at 6000 rpm for 15 min. The glass output coupler was placed at the center of a rotating plate with a speed of 300 rpm, and 20 µL of MoWS_2_ nanosheet liquid supernatant was spin-coated onto its surface. Finally, MoWS_2_ SA samples were obtained by drying them at ambient temperature.

**Figure 1:**
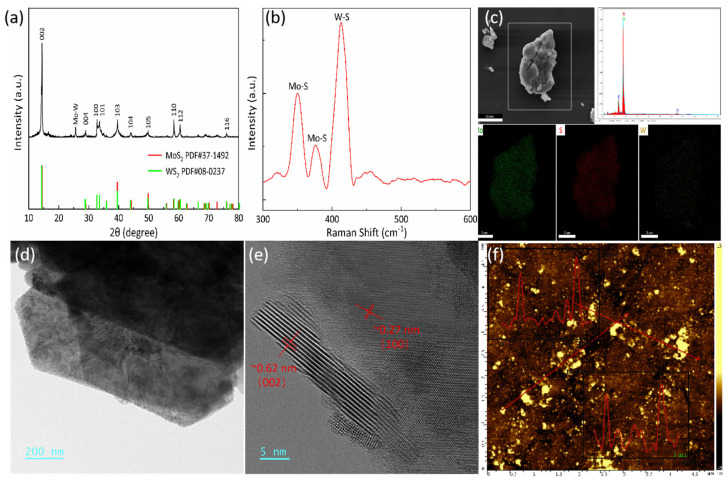
Characterization results of the structure and morphology: (**a**) XRD spectra, (**b**) Raman spectrum, (**c**) SEM and EDS mapping pattern, (**d**) TEM image, (**e**) high-resolution TEM image, (**f**) AFM image and height distribution.

Crystallographic characteristics of the MoWS2 crystal were analyzed using X-ray diffraction (XRD). As shown in Figure 1a, the phase structure investigation confirms that the sample comprises a single-phase 2H-type material [22], aligning with previous studies [23]. Furthermore, Raman spectrum analysis was conducted on the synthesized MoWS_2_ sample using LabRAM HR Evolution Raman spectroscopy at a wavelength of 532 nm. Figure 1b illustrates the Raman spectrum displaying peaks at 350 cm^−1^, 375.5 cm^−1^, and 412.8 cm^−1^, with full width at half maximum (FWHM) values of 14.6, 15.7, and 16.6 cm^−1^, respectively. The first two peaks correspond to the E^1^_2g_ and A^1^_g_ modes, respectively. Typically, the standard Mo-S structure exhibits peaks at 407 and 374 cm^−1^ [24]. However, upon W-atom doping, observed peak shifts occur, including the emergence of a third peak at 412.8 cm^−1^, indicating the presence of the W-S structure. The scanning electron microscope (SEM) and energy dispersive spectrometer (EDS) mapping patterns in Figure 1c further validate the uniform distribution of the three components. Additionally, morphological images reveal the material’s layered nanostructure properties. Figure 1d,e presents transmission electron microscope (TEM) images at different size scales. These images distinctly display nanostructures with an interlayer spacing of 0.62 nm, clearly visible in the high-resolution TEM image. The observed distance of 0.27 nm corresponds to the (100) plane of MoWS_2_, similar to MoS_2_ or WS_2_, suggesting lattice structures akin to those found in these materials [25]. The dimensions of the synthesized MoWS_2_ were further characterized using atomic force microscopy (AFM), depicted in Figure 1f. The material exhibited a thickness of approximately 10 nm with a lateral size of ~µm. It can be concluded that few-layer MoWS_2_ nanosheets are successfully exfoliated using the liquid-phase exfoliation method.

**Figure 2:**
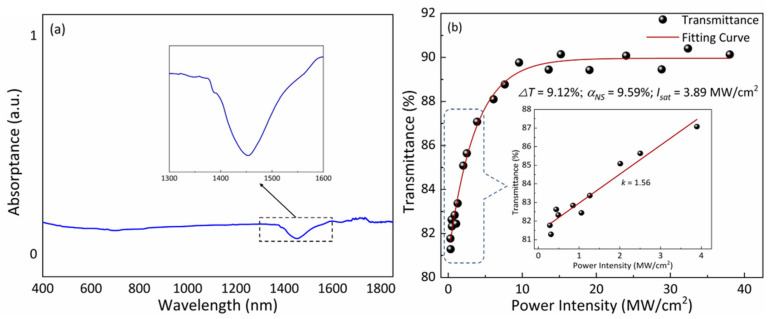
(**a**) Absorption spectrum and (**b**) nonlinear transmittance curve of the MoWS_2_ SA (inset: the linear relation for low-power density).

The optical responses of MoWS_2_ are delineated in Figure 2. Utilizing a UV–Vis–NIR spectrophotometer, the MoWS_2_ SA demonstrated consistent broadband optical absorption across a wavelength spectrum ranging from 400 to 1850 nm. However, a notable reduction in the absorption spectrum was observed specifically in the wavelength range from 1300 to 1600 nm, as portrayed in the inset in Figure 2a. At sufficiently high excitation levels, the MoWS_2_ SA showcased intensity-dependent transmittance due to the Pauli blocking principle. To elucidate the nonlinear absorption characteristics of MoWS_2_ SA, a balanced twin detector experiment was conducted using a custom-built Nd-doped Q-switching laser source with a 50 ns pulse width. This experimental setup allows the derivation of parameters such as the non-saturable absorption loss $\alpha_{NS}$, the modulation depth ΔT, and the saturated optical intensity $I_{sat}$, which can be computed using the following formula [26]:

(1) $$T=1-\alpha_{NS}-\Delta T\exp(-\frac{I}{I_{sat}}),$$

The nonlinear parameter at a specific position encapsulates the collective effect of numerous MoWS_2_ nanosheets with varying thicknesses within the irradiation spot, spanning approximately hundreds of microns. By fitting the experimental data of the nonlinear transmittance showcased in Figure 2b, the intensity-independent $\alpha_{NS}$ is determined to be 9.59%. Additionally, the ΔT is estimated to be 9.12%, while the $I_{sat}$ is approximately 3.89 MW/cm^2^. The inset in Figure 2b exhibits a linear relationship between transmittance and peak-energy density at low-power levels, where a slope of 1.56 is obtained through linear regression. These characteristics highlight the promising potential of MoWS_2_ SA in generating laser pulses. The MoWS_2_ SA used in this study had a thickness of approximately 10 nm. Despite the significant absorption observed, the high damage threshold of MoWS_2_ SA, combined with appropriate thermal management and operation within safe pump intensities, ensures the longevity of the SA without significant risk of long-term damage.

Given that the ground-state population density $n_{s0}$, the cross-sections of the excited state absorption (ESA) $\sigma_{e}$, and the ground-state absorption (GSA) $\sigma_{g}$ constitute the sources of the small-signal transmittance $T_{0}$ and the maximum transmittance $T_{\max}$ actions, the correlation parameters of MoWS_2_ SA can be determined using the following equations [8]:

(2) $$\frac{\sigma_{g}}{\sigma_{e}}=\frac{\ln T_{0}}{\ln T_{\max}},$$

(3) $$\frac{(\sigma_{g}-\sigma_{e})\times T_{0}}{hv}=k,$$

(4) $$T_{0}=\exp(-n_{s0}\sigma_{g}l_{sa}),$$

(5) $$T_{\max}=\exp(-n_{s0}\sigma_{e}l_{sa}),$$

where h stands for the Planck constant and $l_{sa}$ is the thickness of MoWS_2_ SA (~10 nm). According to the information available in Figure 2b, the $T_{0}$ at low power density, the $T_{\max}$ at high power density, and the slope *k* can be discerned, and the calculated results indicate that $\sigma_{g}$ is 6.98 × 10^−19^ cm^2^, $\sigma_{e}$ is 3.4 × 10^−19^ cm^2^, and $n_{s0}$ is 2.968 × 10^23^ cm^−3^, respectively. Considering the inhomogeneous broadening mechanism, it is determined that the excited-state lifetime $\tau_{s}$ of the MoWS_2_ SA is 714.1 µs [8]. Table 1 presents the key parameters concerning the saturable absorption characteristics of 2D MoWS_2_ SA.

### 2.2. Experimental Setup

**Figure 3:**
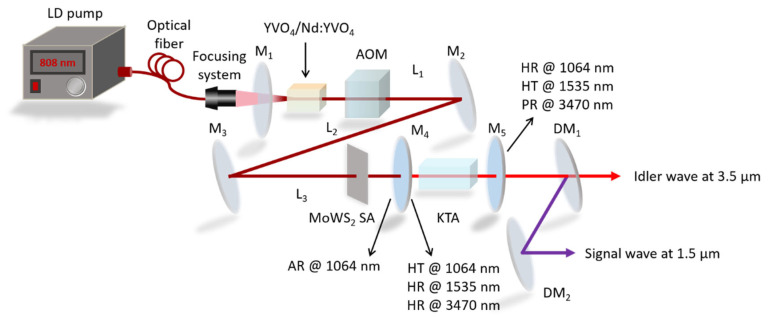
Schematic diagram of the experimental setup.

Figure 3 depicts the schematic of the experimental setup. Considering thermal lensing, cavity stability, and fundamental beam waist concerns, we designed a linear Z-cavity based on the ABCD transmission matrix theory [27]. We adopted a Z-type folded cavity for the fundamental wave cavity, with a length of 119.5 cm, alongside an idler-resonant OPO cavity length of 3.8 cm. The laser system was powered by a commercial fiber-coupled diode laser (FAP system, COHERENT Inc., Saxonburg, PA, USA) at a central wavelength of 808 nm. An optical re-imaging system focused the diode laser into the laser crystal, generating a pump beam with a beam radius around 200 μm. An a-cut composite crystal YVO_4_/Nd:YVO_4_ with dimensions of 3 mm × 3 mm × (2 + 10) mm and Nd-doping concentration of 0.3 at% was utilized as the laser medium. Both surfaces of the YVO_4_/Nd:YVO_4_ crystal were polished and anti-reflection (AR)-coated at 808 and 1064 nm. The nonlinear crystal KTA with dimensions of 5 mm × 5 mm × 20 mm was cut for type-II noncritical phase-matching configuration (θ = 90°, ϕ = 0°) for optimal effective nonlinear coefficient and acceptance angle and AR-coated at 1064 and 1535 nm (R < 0.2%) and high-transmission (HT)-coated at 3470 nm (T > 95%) on both sides. A 47-mm-long AOM (GSQ27-3, central frequency: 27.12 MHz, RF power: 50 W, 26th Institute, CETC, Beijing, China) with AR-coated at 1064 nm (R < 0.2%) on both surfaces served as the active modulator, capable of modulation repetition rates from 1 to 50 kHz. To manage heat dispersion, the laser medium and KTA crystals were wrapped with a thin layer of indium foil, placed in copper holders, and cooled via a thermoelectric cooler at 16 °C for stable laser output. The as-prepared MoWS_2_ was employed as the SA in the fundamental laser system. The lengths of the three cavity arms (L_1_, L_2_, and L_3_) were 40.2, 68.5, and 10.8 cm, respectively, corresponding to a fundamental wave’s round-trip time of approximately 8.26 ns. Optimizing the cavity length was crucial to minimize the number of ML pulses within a Q-switched envelope. Longer cavities reduced the number of ML pulses but broadened the Q-switched envelope, necessitating theoretical and experimental optimization. As the input mirror, the plane mirror M_1_ was AR-coated at 808 nm (R < 0.2%) on its entrance surface, and high-reflectivity (HR)-coated at 1064 nm (R > 99.8%) and HT-coated at 808 nm (T > 97%) on the opposite surface. The folded concave mirrors M_2_, with a radius of curvature (ROC) of 500 mm, and M_3_, with a ROC of 200 mm, were HR-coated at 1064 nm. The flat mirror M_4_ (JGS3) was AR-coated at 1064 nm (R < 0.2%) on its entrance surface, and HT-coated at 1064 nm (T > 99.5%) and HR-coated at both 1535 and 3470 nm (R > 99.8%) on the opposite surface. The flat mirror M_5_ (Al_2_O_3_) was HR-coated at 1064 nm (R > 99.9%), HT-coated at 1535 nm (T > 99.3%), and partial-reflectivity (PR)-coated at 3470 nm (R = 91%). Thus, the idler-resonant OPO cavity oscillated between M_4_ and M_5_, while the fundamental wave oscillated between M_1_ and M_5_.

To distinguish between the idler, signal, and residual fundamental waves, optical components were strategically selected. A dichroic mirror DM_1_ (HR-coated at both 1535 and 1064 nm and HT-coated at 3470 nm) crafted from CaF_2_ was utilized to effectively separate the idler wave. Meanwhile, a dichroic mirror DM_2_ (HR-coated at 1535 nm and HT-coated at 1064 nm) made of BK7 glass was employed to differentiate the signal wave from the residual fundamental wave. Precise measurement tools were employed to assess pulse characteristics and wave spectra. A DPO-7104C digital oscilloscope (rise time: 350 ps, 1 GHz bandwidth, 20 G samples/s, Tektronix Inc., Beaverton, OR, USA), a fast InGaAs photodetector with a rising time of 0.4 ns (New Focus, 1611) and a Wavescan laser spectrometer (resolution: 0.4 nm, APE GmbH, Berlin, Germany) were employed to measure the pulse characteristics and the spectrum of the signal wave. The pulse characteristics of the idler wave were acquired by the DPO-7104C digital oscilloscope and an HgCdZnTe photoconductive detector with a response time of 1 ns. The idler wave spectra were measured by another Wavescan MIR laser spectrometer (APE GmbH, Berlin, Germany). Precise measurement of output powers was conducted using a PM100D laser power meter from Thorlabs Inc. (Newton, NJ, USA) to quantify the output powers of both the signal and idler waves.

## 3. Results and Discussion

**Figure 4:**
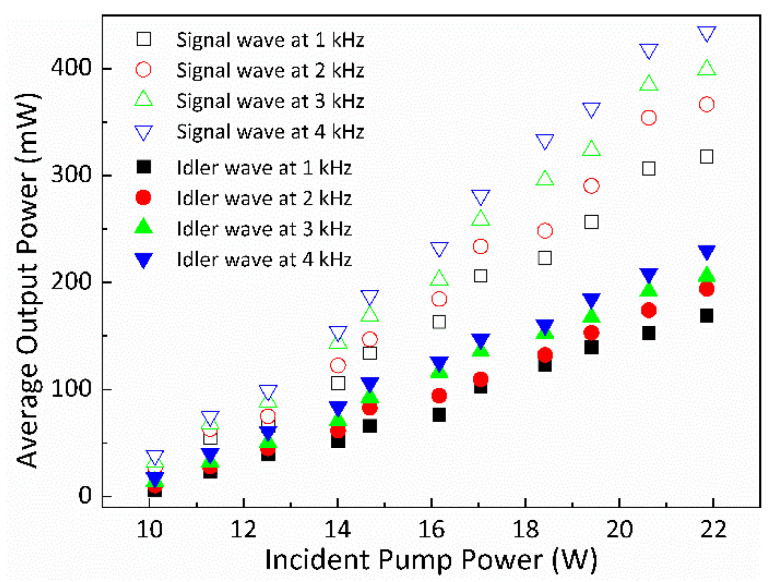
The average output powers of the signal and idler waves versus pump powers for different $f_{p}$.

In the experiment, the diode-pumped idler-resonant dual-loss-modulated QML YVO_4_/Nd:YVO_4_/KTA IOPO system operates in two distinct stages: the QML stage and the sub-nanosecond single mode-locking (ML) stage. The experimental results indicated that the primary factors influencing the OPO’s operational status were the active modulation frequency and pump power. The repetition rates of the Q-switched envelopes were controlled by the AOM. During the QML stage, the pulse width of the Q-switched envelope and the number of mode-locking pulses within the Q-switched envelope decrease with increasing pump power and decreasing AOM repetition rate. When the pulse width of the Q-switched envelope becomes shorter than the time interval between two mode-locking pulses, only one mode-locking pulse exists within a Q-switched envelope, marking the transition to the sub-nanosecond single ML stage. At this stage, the OPO output pulses are sub-nanosecond with repetition rates determined by the AOM. The threshold power required for the signal and idler waves was approximately 10.1 W. Figure 4 depicts the average output powers of both the signal and idler waves concerning the pump powers across various modulation frequencies $f_{p}$. A clear trend is evident, showcasing a consistent rise in the average output powers of both waves with increasing incident pump powers and modulation frequencies. At the point where the pump power reached 21.8 W, the maximum average output powers for the signal wave, observed at modulation frequencies of 1, 2, 3, and 4 kHz, were 318, 364, 398, and 434 mW, respectively. Simultaneously, the corresponding maximum average output powers for the idler wave stood at 169, 194, 206, and 230 mW for the same frequencies.

**Figure 5:**
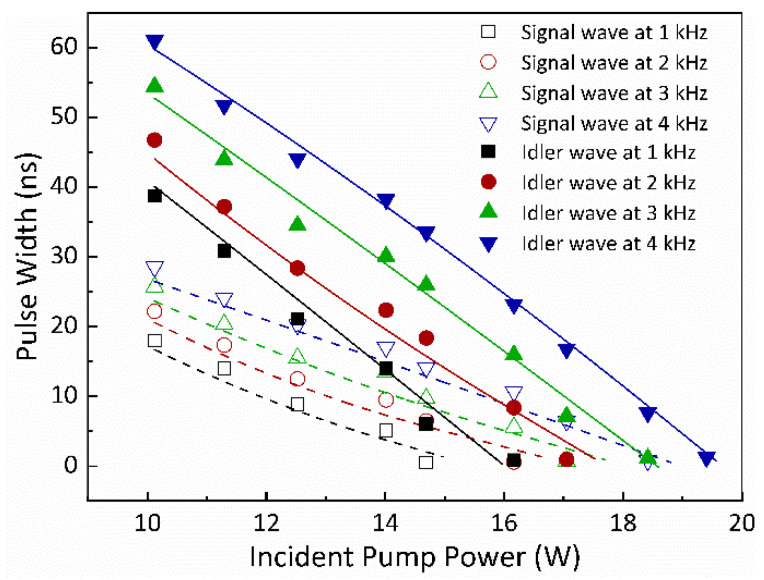
The pulse widths of the Q-switched envelopes for the signal and idler waves versus pump powers for different $f_{p}$. Symbol: experimental data; curve: theoretical result.

The experimental results, depicted in Figure 5 using scattered symbols, showcased the durations of the signal and idler waves’ Q-switched envelopes concerning various pump powers for different modulation frequencies $f_{p}$. For the signal wave’s single ML pulse generation, the experimentally determined threshold powers were 14.68 W for $f_{p}$ = 1 kHz, 16.16 W for $f_{p}$ = 2 kHz, 17.05 W for $f_{p}$ = 3 kHz, and 18.41 W for $f_{p}$ = 4 kHz. Concurrently, the threshold powers for the idler wave’s single ML pulse generation were 16.16 W for $f_{p}$ = 1 kHz, 17.05 W for $f_{p}$ = 2 kHz, 18.41 W for $f_{p}$ = 3 kHz, and 19.4 W for $f_{p}$ = 4 kHz. These specific pump powers represent the threshold values for the generation of single ML pulses for the signal and idler waves. It is important to note that the idler-resonant KTA IOPO driven by the dual-loss-modulated QML laser can generate sub-nanosecond single ML pulses of the signal and idler waves only when the pump powers surpass these threshold values. Notably, at equivalent modulation frequencies $f_{p}$, the threshold power for generating the idler wave’s single ML pulse was marginally higher than that required for the signal wave’s single ML pulse.

**Figure 6:**
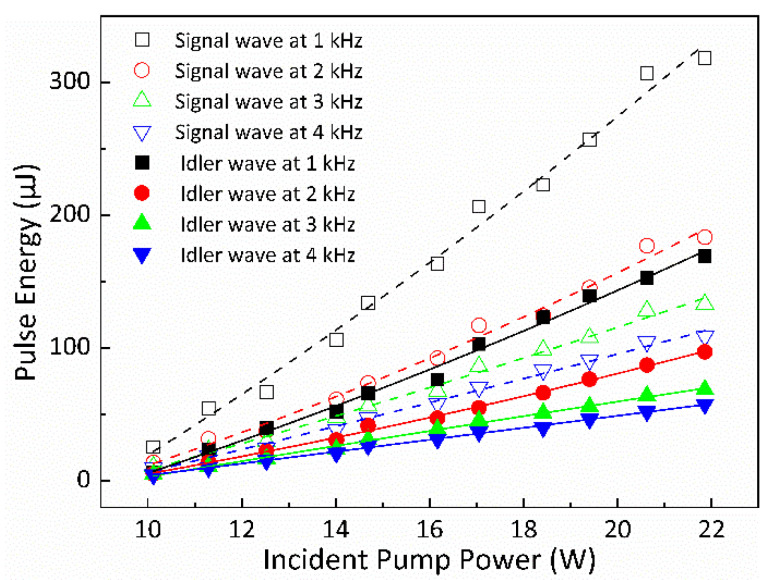
The pulse energies of the signal and idler waves’ Q-switched pulses versus pump powers for different $f_{p}$. Symbol: experimental data; curve: theoretical result.

**Figure 7:**
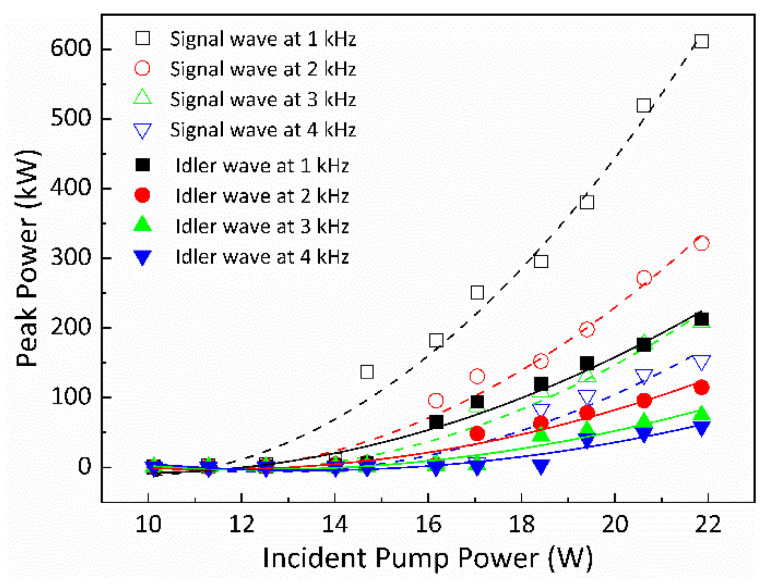
The peak powers of the signal and idler waves’ Q-switched pulses versus pump powers for different $f_{p}$. Symbol: experimental data; curve: theoretical result.

The pulse energies of the signal and idler waves’ Q-switched envelopes for different $f_{p}$ were determined based on the average output powers and the pulse repetition rates, and the results are depicted in Figure 6 using scattered symbols. Specifically, the maximum pulse energies for the signal wave were found to be 318, 182, 132.6, and 108.5 μJ under modulation frequencies of 1, 2, 3, and 4 kHz, respectively. Similarly, for the idler wave, the maximum pulse energies were 169, 97, 68.6, and 57.5 μJ, respectively, at the corresponding frequencies. Hence, it can be inferred that lower repetition rates contribute to enhancing the energies of both the signal and idler waves’ single ML pulses. By employing the durations and energies of the single ML pulses for the signal and idler waves, the estimated peak powers for the signal wave’s single ML pulses were calculated as 441.6, 242.6, 170.1, and 132.3 kW for modulation frequencies of 1, 2, 3, and 4 kHz, respectively. Similarly, the estimated peak powers for the idler wave’s single ML pulses were calculated as 203.6, 111.5, 74.6, and 59.9 kW for the respective modulation frequencies of 1, 2, 3, and 4 kHz, as seen in Figure 7 with scattered symbols.

**Figure 8:**
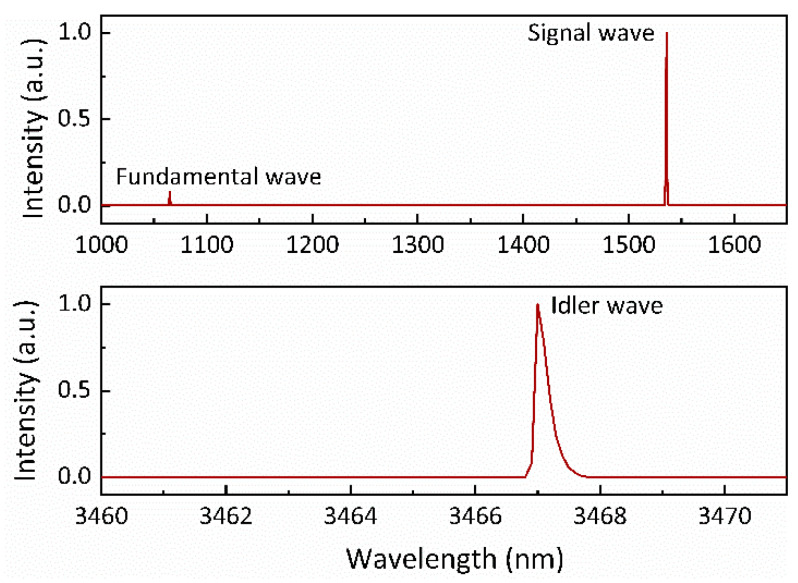
A representative output spectrum of the idler-resonant dual-loss-modulated QML KTA IOPO at an incident pump power of 19.4 W and an AOM modulation rate of 1 kHz.

Figure 8 presents the typical output spectrum of the idler-resonant dual-loss-modulated QML KTA IOPO at an incident pump power of 19.4 W and an AOM modulation rate of 1 kHz. The spectrum exhibited the fundamental and signal waves at 1064 nm and 1535 nm, respectively, while the idler wave was detected at a wavelength of 3467 nm.

**Figure 9:**
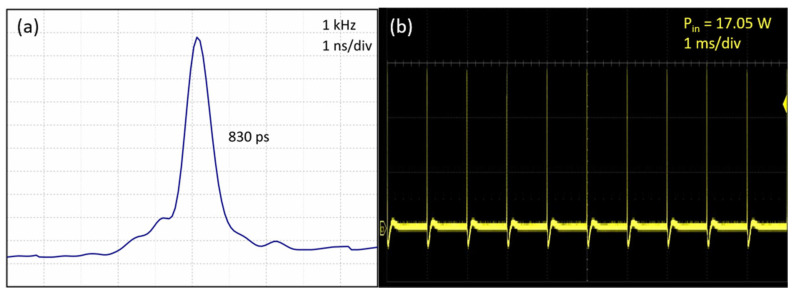
(**a**) Extended temporal profile of the idler wave’s sub-nanosecond single ML pulse for $f_{p}$ = 1 kHz and (**b**) oscilloscope trace of the idler wave’s single ML pulse train for $f_{p}$ = 1 kHz.

In Figure 9a, an extended temporal profile of the idler wave’s sub-nanosecond single ML pulse at $f_{p}$ = 1 kHz for the pump power of 16.16 W is depicted. The extended oscilloscope trace enables estimation of the ML pulse width, as shown in Figure 9a [28,29]. Based on this trace, the estimated single ML pulse width was approximately 830 ps, corresponding closely to $\tau_{p}$ = 471 ps. Moreover, to illustrate the stability of the single ML pulse IOPO laser, a temporal idler wave’s single ML pulse train, recorded by the digital oscilloscope for $f_{p}$ = 1 kHz at the pump power of 17.05 W, is displayed in Figure 9b. The pulse-to-pulse amplitude fluctuation, defined as the ratio between the highest deviation and the mean pulse amplitude, measured within the 1 kHz sub-nanosecond idler wave laser pulses, was found to be less than 4%. This result underscores the high stability of the laser output.

**Figure 10:**
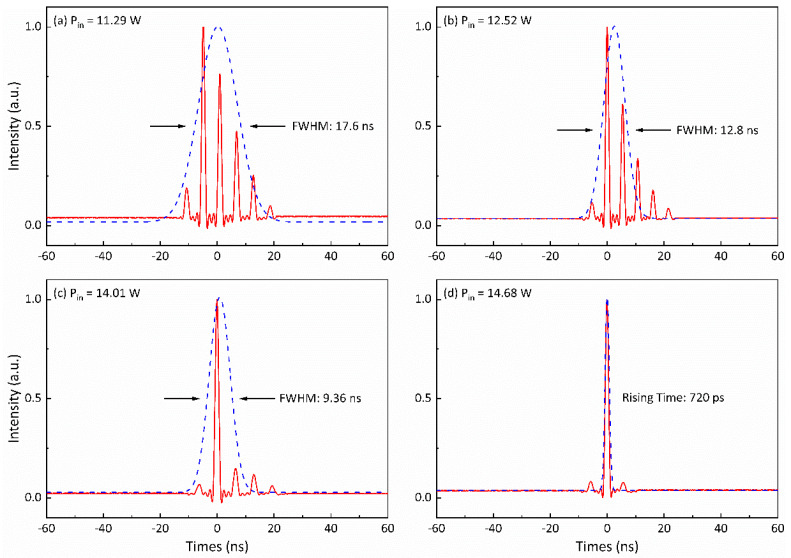
The pulse shapes of the signal wave at various incident pump powers when the modulation frequency is 1 kHz: (**a**) 11.29 W, (**b**) 12.52 W, (**c**) 14.01 W, (**d**) 14.68 W.

**Figure 11:**
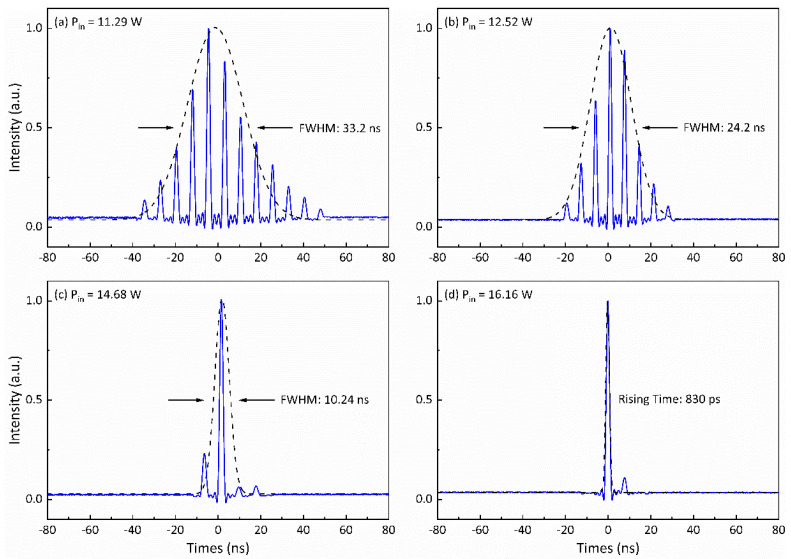
The pulse shapes of the idler wave at different incident pump powers when the modulation frequency is 1 kHz: (**a**) 11.29 W, (**b**) 12.52 W, (**c**) 14.68 W, (**d**) 16.16 W.

Figure 10 and Figure 11 show the oscilloscope traces illustrating the pulses of the signal and idler waves within the idler-resonant dual-loss-modulated QML KTA IOPO at various incident pump powers, while maintaining the AOM’s modulated frequency at 1 kHz, respectively. In particular, observations from Figure 10a–d demonstrate a noticeable trend: The pulse widths of the signal wave’s Q-switched envelopes progressively diminish with increased incident pump power. This reduction results in a corresponding decrease in the number of ML pulses present underneath the Q-switched envelope. Specifically, at pump powers of 11.29 W, 12.52 W, and 14.01 W, there were five, three, and two ML pulses, respectively, coexisting beneath the signal wave’s Q-switched envelope. Remarkably, upon reaching 14.68 W pump power (Figure 10d), it is evident that a single sub-nanosecond ML pulse beneath the Q-switched envelope of the signal wave was successfully achieved.

Furthermore, as showcased in Figure 11, the pulse widths of the idler wave’s Q-switched envelopes decreased consistently with increasing incident pump power. Specifically, at pump powers of 11.29 W, 12.52 W, and 14.68 W, there were eight, six, and two ML pulses, respectively, coexisting beneath the idler wave’s Q-switched envelope. Notably, upon reaching 16.16 W pump power (Figure 11d), it becomes apparent that a single sub-nanosecond ML pulse beneath the idler wave’s Q-switched envelope was successfully achieved.

Additionally, wavelength-tuning in our OPO setup is achieved primarily by adjusting the OPO or using a tunable pump source. In the case of our idler-resonant intracavity KTA OPO, the tuning of the output wavelength is controlled by varying the phase-matching conditions within the nonlinear KTA crystal. This can be realized mainly by temperature tuning or angle tuning. This allows for fine adjustments to the signal and idler wavelengths, thereby enabling tunability of the output wavelength. Additionally, tuning can be achieved by altering the pump power or the cavity configuration, which impacts the gain and resonant conditions of the OPO system. These methods enable broad spectral tunability while maintaining the performance of the 2D MoWS_2_ SA. In our experiments, we observed that the pulse widths of the signal and idler waves varied with tuning wavelength due to the wavelength-dependent nonlinear optical properties of the MoWS_2_ SA. As the wavelength is tuned, changes in the absorption cross-section and saturation intensity influence the mode-locking dynamics, leading to different pulse durations. Specifically, at shorter wavelengths, the stronger absorption characteristics of MoWS_2_ lead to shorter pulse widths due to more efficient mode-locking. Conversely, at longer wavelengths, where the absorption is weaker, the pulse width tends to increase.

## 4. Theoretical Analysis

In an effort to analyze the dynamic mechanism of the diode-pumped idler-resonant KTA IOPO driven by a doubly QML YVO_4_/Nd:YVO_4_ laser with AOM and MoWS_2_ SA, the coupled rate equations can be utilized to obtain the corresponding characteristics. Based on the fluctuation mechanism [30,31], it is observed that the ML pulse shape remains stable after multiple round-trips within the laser cavity. Employing the assumption of Gaussian spatial distribution, the intracavity photon density corresponding to the TEM_00_ mode of the fundamental wave can be mathematically described as per [32]:

(6) $$\varphi (r,t)=\sum_{k=0}\Phi_{k}f(t-t_{k})\exp(-\frac{2r^{2}}{w_{l}^{2}})=\varphi (0,t)\exp(-\frac{2r^{2}}{w_{l}^{2}}),$$

where $\varphi (0,t)=\sum_{k=0}\Phi_{k}f(t-t_{k})$ represents the photon density along the laser axis, with $w_{l}$ being the average radius of the TEM_00_ mode oscillating laser within the cavity. Here, *r* stands for the radial coordinate, *t* refers to time, and $\Phi_{k}$ denotes the relative amplitude of the ML pulses during the *k*th roundtrip. Additionally, $f(t)=\frac{1}{2\sigma c\tau_{p}}{\mathrm{sech}}^{2}(\frac{t}{\tau_{p}})$ signifies the onset of ML pulses evolving from noise. In relation to the resonator round-trip transit time *t_r_*, f(t) is assumed to be a sharp pulse centered at *t* = 0, exhibiting a rapid decay within a short duration. In this context, $t_{r}=2(n_{g}l+n_{sa}l_{sa}+n_{a}l_{a}+n_{k}l_{KTA}+L_{c}-l-l_{sa}-l_{a}-l_{KTA})/c$ stands for the round-trip time within the fundamental cavity, where $n_{g}$, $n_{sa}$, $n_{a}$, and $n_{k}$ denote the refractive indices of the gain medium, MoWS_2_ SA, AOM crystal, and KTA crystal, respectively. The variables l, $l_{sa}$, $l_{a}$, and $l_{KTA}$ represent the lengths of the gain medium, MoWS_2_ SA, AOM crystal, and KTA crystal, while $L_{c}$ denotes the physical length of the fundamental wave cavity. c stands for the speed of light in vacuum, and $\tau_{p}$ correlates to the FWHM of the ML pulse duration at the fundamental wavelength through the equation $\tau =1.76\tau_{p}$.

Therefore, the temporal photon intensity at different positions within the cavity during the *k*th roundtrip can be described as follows:

(7) $$\varphi_{k,i}=\frac{w_{l}^{2}}{w_{i}^{2}}\exp(-\frac{2r^{2}}{w_{i}^{2}})\Phi_{k}f(t),\;(i\;=\;g,\;s,\;a,\;k)$$

where $w_{i}$ symbolizes the average beam radii of the TEM_00_ mode positioned at the gain medium, MoWS_2_ SA, AOM crystal, and KTA crystal, respectively. These values can be computed utilizing the ABCD matrix theory.

### 4.1. Influence of AOM

As an active modulator, an AOM serves to mitigate its intrinsic statistical nature during the initial stages of pulse generation while retaining the nonlinear attributes of the saturable absorber and the Q-switch during the later stages of pulse compression. In each laser shot, the statistical noise pulse, which eventually becomes the ML pulse, undergoes modulation by the active modulator, resulting in an improved stability of pulse energy from shot to shot. The loss function of the AOM can be denoted as $\delta_{a}(t)=\delta_{a}\exp(-t^{2}/t_{ao}^{2})$, where $\delta_{a}$ represents the inherent diffraction loss of the AOM, and $t_{ao}$ signifies the turnoff time of the AOM.

Given that the repetition rate of the Q-switched envelopes is reliant on the modulation frequency of the AOM, the initial population inversion density of the laser crystal can be derived. Preceding the onset of a laser pulse, the initial population density at the lower laser level is assumed to be zero for a four-level gain medium. Thus, considering the Boltzmann distribution coefficient *f_a_* in the upper level, the initial population inversion density is expressed as follows [32]:

(8) $$n_{initial}=f_{a}R_{in}\tau_{m}\left[1-\exp(-\frac{1}{f_{p}\tau_{m}})\right],$$

where $f_{p}$ denotes the modulation frequency of the AOM and $\tau_{m}$ represents the lifetime of the upper laser level of the laser medium. Additionally, $R_{in}(r)=\frac{P_{in}\exp(-2r^{2}/w_{in}^{2})\left[1-\exp(-\alpha l)\right]}{hv_{in}\pi w_{in}^{2}l}$ signifies the pump rate, where $P_{in}$ is the pump power, $hv_{in}$ denotes the single-photon energy of the pump light, $w_{in}$ represents the average radius of the pump beam, and α signifies the absorption coefficient of the gain medium.

### 4.2. Signal Wave’s Photon Density

In our idler-resonant IOPO, it functions as a pure singly-resonant oscillator (SRO) where the output coupler remains perfectly transparent for the signal wave. This signifies that the signal wave does not undergo the cavity. Similarly, in the context of most second harmonic generation (SHG) processes, the frequency-doubled laser also experiences no cavity, akin to the behavior of the signal wave in our IOPO. Hence, the dynamic process governing the signal wave generation in the idler-resonant IOPO is applicable when considering the SHG process of the frequency-doubling laser. Under these analogous scenarios, the photon density of the signal wave can be formulated as follows [33]:

(9) $$\varphi_{s}(0,t)=\frac{ћ\mu_{0}d_{eff}^{2}\omega_{f}\omega_{s}\omega_{id}}{4n_{f}^{2}n_{i}^{2}}{\left(2l_{KTA}\right)}^{2}\varphi (0,t)\varphi_{id}(0,t),$$

where $\mu_{0}$ denotes the permeability of vacuum, while $\omega_{f,s,id}$ represent the circular frequencies corresponding to the fundamental, signal, and idler waves, respectively. The effective nonlinear coefficient is symbolized as *d_eff_*, whereas $n_{f,i}$ stand for the refractive indices of the fundamental and idler waves in the KTA medium, respectively.

### 4.3. Rate Equations

The interaction between the fundamental and idler waves exclusively took place within the OPO cavity. To reflect this, we introduced a gate function that altered the coupled rate equations:

(10) $$G(t)=\left\{\begin{array}{l} 1,t_{k}<t<t_{k}+t_{rs}\\ 0,t_{k}+t_{rs}<t<t_{k+1} \end{array}\right.,$$

Simultaneously, considering the transmission surpassing 99.3% at the signal wave by the output coupler (M_5_), it effectively arrested the evolutionary process of the signal wave field within the idler-resonant IOPO. Given this aspect, the coupled rate equations governing the idler-resonant IOPO are formulated by amalgamating the rate equations characterizing the diode-pumped dual-loss-modulated QML laser involving AOM and MoWS_2_ SA [31] with the equations relevant to the IOPOs [34]:

(11) $$\frac{d\varphi (r,t)}{dt}=\int \frac{1}{t_{r}}\left\{2\sigma n(r,t)l\varphi_{g}(r,t)\right.-2\sigma_{g}n_{s1}(r,t)l_{s}\varphi_{s}(r,t)-2\sigma_{e}\left[n_{s0}-n_{s1}(r,t)\right]l_{s}\varphi_{s}(r,t)\;\left.-\delta_{a}(t)\varphi_{a}(r,t)-L\varphi (r,t)-2\sigma_{opo}cl_{KTA}\varphi_{k}(r,t)\varphi_{id}(r,t)\right\}2\pi rdr,$$

(12) $$\frac{dn(r,t)}{dt}=R_{in}(r)-\sigma cn(r,t)\varphi_{g}(r,t)-\frac{n(r,t)}{\tau },$$

(13) $$\frac{dn_{s1}(r,t)}{dt}=\frac{n_{s0}-n_{s1}(r,t)}{\tau_{s}}-\sigma_{g}cn_{s1}(r,t)\varphi_{s}(r,t),$$

(14) $$\frac{d\varphi_{id}(r,t)}{dt}=\int \frac{1}{t_{rid}}\left\{2\sigma_{opo}cl_{KTA}\varphi_{k}(r,t)\varphi_{id}(r,t)\right.G(t)\left.-\left[L_{id}+\ln\left(\frac{1}{R_{id}}\right)\right]\varphi_{id}(r,t)\right\}2\pi rdr,$$

where n(r,t) denotes the average population-inversion density, while σ represents the stimulated emission cross-section of the gain medium. Additionally, $\sigma_{g}$ and $\sigma_{e}$ stand for the cross-sections related to the GSA and ESA of the MoWS_2_ SA. Moreover, $n_{s0}$ and $n_{s1}(r,t)$ signify the population densities pertaining to the ground-state and the excited-state of the MoWS_2_ SA. The variables $\varphi_{i}(r,t)$ (*i* = *g*, *s*, *a*, *k*) indicate the photon densities located at the positions of the gain medium, MoWS_2_ SA, AOM crystal, and KTA crystal, respectively, obtainable through the utilization of Equation (7). Furthermore, $\varphi_{id}(r,t)$ symbolizes the idler wave photon density, whereas $\sigma_{opo}=2\pi^{2}d_{eff}^{2}hv_{f}v_{s}v_{i}l_{KTA}/\left(\varepsilon_{0}n_{f}^{2}n_{i}^{2}n_{s}l_{f}c^{2}\right)$ represents the parametric gain coefficient, with $v_{f,s,i}$ being the frequencies of the fundamental, signal, and idler waves, respectively. Moreover, $n_{s}$ denotes the refractive index of the signal wave in the KTA crystal. L represents the intrinsic loss for the fundamental wave, while τ stands for the stimulated-radiation lifetime of the gain medium. Additionally, $\tau_{s}$ and $t_{rid}$ correspond to the excited-state lifetime of the MoWS_2_ SA and the round-trip time of the idler wave, respectively. Furthermore, $L_{id}$ denotes the intrinsic loss of the idler wave, and $R_{id}$ stands for the reflectivity at the idler wave of the output coupler.

To simplify, we make the assumption that the modes of the fundamental and idler waves inside the KTA crystal exhibit significant overlap, i.e., $w_{k}=w_{id}$. Under this particular condition, at the *k*th round-trip time $t_{k}=kt_{r}$, the rate equations can be further articulated as:

(15) $$\Phi_{k}=\Phi_{k-1}\exp\left\{\frac{2}{\pi w_{l}^{2}}\int \left[2\sigma n(r,t_{k})\right.\right.l\frac{w_{l}^{2}}{w_{g}^{2}}\exp\left(-\frac{2r^{2}}{w_{g}^{2}}\right)\;-2\sigma_{g}n_{s1}(r,t_{k})l_{sa}\frac{w_{l}^{2}}{w_{s}^{2}}\exp\left(-\frac{2r^{2}}{w_{s}^{2}}\right)\;-2\sigma_{e}\left(n_{s0}-n_{s1}(r,t_{k})\right)l_{sa}\frac{w_{l}^{2}}{w_{s}^{2}}\exp\left(-\frac{2r^{2}}{w_{s}^{2}}\right)\;-\delta_{a}\frac{w_{l}^{2}}{w_{a}^{2}}\exp\left(-\frac{2r^{2}}{w_{a}^{2}}\right)-Lxp\left(-\frac{2r^{2}}{w_{l}^{2}}\right)\;\left.\left.-2\sigma_{opo}cl_{KTA}\frac{w_{l}^{2}}{w_{k}^{2}}\phi_{id}(0,t_{k})\exp\left(-\frac{4r^{2}}{w_{k}^{2}}\right)\right]2\pi r\right\}dr,$$

(16) $$n(r,t_{k})=\exp\left(-\frac{t_{k}}{\tau }\right)\prod_{0}^{k-1}\exp\left[-\frac{w_{l}^{2}}{w_{g}^{2}}\exp\left(-\frac{2r^{2}}{w_{g}^{2}}\right)\Phi_{m}\right]\;\times \left\{R_{in}(r)\exp\left(-\frac{2r^{2}}{w_{p}^{2}}\right)\right.\exp\left(\frac{t_{k}}{\tau }\right)\int_{0}^{t_{k}}\prod_{0}^{k-1}\exp\left[\frac{w_{l}^{2}}{w_{g}^{2}}\exp\left(-\frac{2r^{2}}{w_{g}^{2}}\right)\Phi_{m}\right]dt\;\left.+n(0,0)\exp\left(-\frac{2r^{2}}{w_{p}^{2}}\right)\right\},$$

(17) $$n_{s1}(r,t_{k})=\exp\left(-\frac{t_{k}}{\tau_{s}}\right){\prod_{0}^{k-1}\exp\left[-\frac{w_{l}^{2}}{w_{s}^{2}}\exp\left(-\frac{2r^{2}}{w_{s}^{2}}\right)\Phi_{m}\right]}^{\sigma_{g}/\sigma }\;\left.\times \left\{\frac{n_{s0}}{\tau_{s}}\exp\left(\frac{t_{k}}{\tau }\right){\int_{0}^{t_{k}}\prod_{0}^{k-1}\exp\left[\frac{w_{l}^{2}}{w_{s}^{2}}\exp\left(-\frac{2r^{2}}{w_{s}^{2}}\right)\Phi_{m}\right]}^{-\sigma_{g}/\sigma }dt\right.+n_{s0}\right\},$$

(18) $$\varphi_{id}(0,t_{k})=\exp\left[-\frac{L_{id}+\ln\left(\frac{1}{R_{id}}\right)}{t_{rid}}t_{k}\right]\prod_{m=0}^{k-1}\exp\left[\frac{1}{t_{rid}}2\sigma_{opo}\right.cl_{KTA}\frac{w_{l}^{2}}{w_{k}^{2}}\left.\exp\left(-\frac{2r^{2}}{w_{k}^{2}}\right)G(t_{m})\Phi_{m}\right],$$

where $w_{p}$ represents the pump beam radius. To define the initial conditions:

(19) $$n(r,0)=n(0,0)\exp(-\frac{2r^{2}}{w_{p}^{2}}),$$

(20) $$n_{s1}(r,0)=n_{s0},$$

By numerically solving Equations (9) and (15)–(20), the resulting output pulse energies for both the idler and signal waves can be derived as follows:

(21) $$E_{id}=\frac{\pi w_{k}^{2}chv_{id}}{4}\ln(\frac{1}{R_{id}})\int_{0}^{\infty }\phi_{id}(0,t)dt,$$

(22) $$E_{s}=\frac{\pi w_{k}^{2}chv_{s}}{4}\int_{0}^{\infty }\phi_{s}(0,t)dt,$$

### 4.4. Numerical Simulation Results

In the numerical simulation, $\tau_{p}$ holds a significant role as it correlates with the ML pulse duration τ. Given the fluctuation mechanism’s assumption that the ML pulse width τ remains constant [32], $\tau_{p}$ can be treated as a constant value throughout the simulation. The numerically simulated value of $\tau_{p}$ is basically in agreement with the experimental duration. It is noteworthy that in the numerical simulation, the pulse width of the ML pulse underneath the Q-switched envelope remained constant, consistent with the fluctuation mechanism’s theoretical explanation. However, in the experimental setup, the pulse width of the ML pulse underneath the Q-switched envelope marginally decreased with increasing pump power. This reduction in pulse width continued as the single ML pulse was generated. In the simulation, the pulse width of the single ML pulse was provided at the threshold pump power to verify the experimental results. Table 2 outlines the values of the other pertinent parameters employed in the numerical simulation. By using the parameters outlined in Table 1 and Table 2, we can ascertain $\Phi_{k}$ with a specified initial value of $\Phi_{0}$ via the numerical solution of Equations (6)–(20). Subsequently, the output pulse energies of the idler and signal waves can be determined using Equations (21) and (22), respectively. Since the average output power within a Q-switched envelope accounts for the summation of the output powers of all the ML pulses under the Q-switched envelope, the pulse width of the Q-switched envelope can be inferred from the shape of *P(t)*. Given that the time interval between two neighboring ML pulses matches the cavity round-trip time, acquiring a single ML pulse beneath a Q-switched envelope necessitates that the pulse width of the Q-switched envelope is shorter than the cavity round-trip time.

The plotted curves in Figure 5 and Figure 6 depict the theoretically calculated data for the widths of the Q-switched envelopes and the pulse energies of the signal and idler waves concerning various pump powers corresponding to different repetition rates of the AOM.

Figure 5 specifically showcases the variations in the pulse widths of the signal and idler waves’ Q-switched envelopes in relation to pump powers for various values of $f_{p}$, represented by different curves. The figure reveals two distinct operational stages of the idler-resonant KTA IOPO driven by a dual-loss-modulated QML laser: the initial QML stage and the final sub-nanosecond single ML stage. During the QML stage, it is evident that the pulse widths of both the signal and idler waves’ Q-switched envelopes decrease as the pump power increases while decreasing $f_{p}$, indicating that lower repetition rates of the AOM lead to shorter pulse durations. At specific pump powers of 14.9 W for $f_{p}$ = 1 kHz, 16.5 W for $f_{p}$ = 2 kHz, 17.6 W for $f_{p}$ = 3 kHz, and 18.7 W for $f_{p}$ = 4 kHz, the pulse widths of the signal wave’s Q-switched envelopes became shorter than the cavity round-trip time, resulting in the generation of a signal wave’s single ML pulse per envelope. Concurrently, when reaching pump powers of 15.9 W for $f_{p}$ = 1 kHz, 17.5 W for $f_{p}$ = 2 kHz, 18.6 W for $f_{p}$ = 3 kHz, and 19.5 W for $f_{p}$ = 4 kHz, the idler wave’s single ML pulse per envelope was achieved. These theoretical calculations were largely consistent with the experimental findings above. However, a minor discrepancy between the experimental and simulation results was observed. Specifically, the experimental threshold powers for achieving the sub-nanosecond single ML pulses of the signal and idler waves were slightly lower than those values by the numerical simulation.

**Figure 12:**
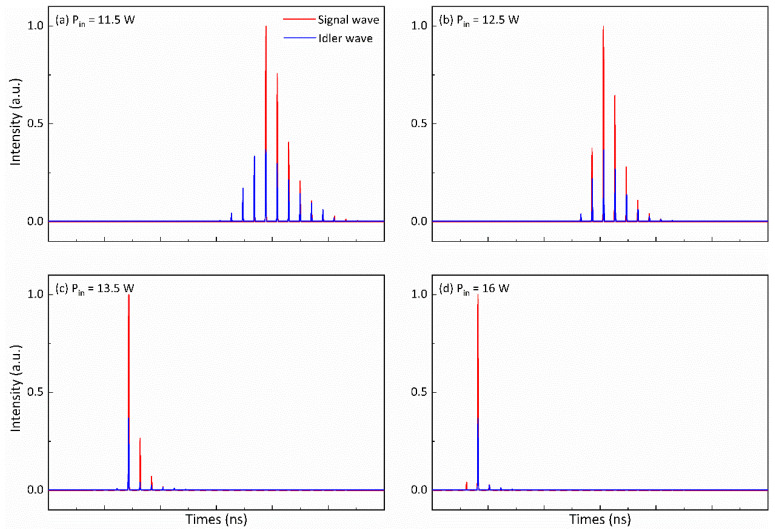
The number of ML pulses within a Q-switched envelope for both the signal and idler waves varies with pump powers at $f_{p}$ = 1 kHz.

When $f_{p}$ = 1 kHz, the variations in the number of ML pulses for both the signal and idler waves within a Q-switched envelope at different pump powers are depicted in Figure 12. From Figure 12a–d, it is evident that the ML pulse count for both the signal and idler waves within a Q-switched envelope decreases with increasing pump power. Notably, when *P_in_* = 16 W, as shown in Figure 12d, there is only a single ML pulse present for both the signal and idler waves beneath the Q-switched envelope. From the results shown in Figure 10, Figure 11 and Figure 12, it can be seen that the theoretical simulation results are consistent with the experimental results, which verifies the correctness of the theoretical simulation.

The simulated pulse energies and peak powers of the signal and idler waves’ Q-switched envelopes for different $f_{p}$ are depicted in Figure 6 and Figure 7 using curves, respectively. The trend shows that the pulse energies of both the signal and idler waves’ Q-switched envelopes exhibit an approximate linear increase in relation to the pump powers. However, it is notable that as $f_{p}$ increases, there is a decrease observed in the pulse energies and peak powers of both the signal and idler waves’ Q-switched envelopes. This suggests that a lower repetition rate tends to enhance the pulse energies and peak powers of the signal and idler waves’ QML pulses.

The performance of 2D nanomaterial-based intracavity OPOs driven by dual-loss-modulated QML lasers at 1 μm wavebands is summarized in Table 3. In comparison to the other 2D nanomaterial-based SAs, the idler wave’s pulse width of the idler-resonant intracavity KTA OPO with MoWS_2_ SA is comparatively shorter, resulting in a relatively high peak power.

The 2D MoWS_2_ SA offers a broad and tunable spectral window, making it highly versatile for various laser applications. In our study, the MoWS_2_ SA demonstrates strong nonlinear optical absorption over a wide wavelength range. By tuning the phase-matching conditions in the intracavity KTA OPO, we can achieve efficient wavelength tunability. This flexibility enables the SA to operate effectively in both the signal and idler wavelengths, showcasing its adaptability for different laser systems. We observed that the MoWS_2_ SA can function across a broad spectral window, from the near-infrared to mid-infrared region, with stable pulse generation. This wideband operation highlights its suitability for wavelength-tunable laser systems. The tunable response, in combination with its high damage threshold, makes the MoWS_2_ SA an ideal candidate for high-power and tunable laser applications. However, stability is critical for practical laser applications, especially in high-power scenarios. Our experimental tests demonstrated that the MoWS_2_ SA maintains consistent performance over extended periods of operation without degradation. The 2D nanomaterial’s robustness under both thermal and optical stress contributes to its long-term stability, even at high power levels. We conducted long-term operation tests and observed no significant changes in the material’s nonlinear properties or damage under high-intensity laser illumination.

## 5. Conclusions

In conclusion, the creation of layered MoWS_2_ through liquid-phase exfoliation methods has showcased exceptional optical modulation properties, particularly for laser pulse generation within the 1 µm wavelength range. The experimental demonstrations confirm the efficiency of the IOPO driven by dual-loss-modulation technology employing AOM and MoWS_2_ SA, highlighting its capability to generate high-peak-power sub-nanosecond near-infrared and mid-infrared single mode-locking pulses. The related coupled rate equations for the diode-pumped, idler-resonant KTA IOPO, driven by a dual-loss-modulated QML YVO_4_/Nd:YVO_4_ laser with AOM and MoWS_2_ SA, have been successfully presented and simulated numerically. Remarkably, these numerical simulation results align fundamentally with the experimental observations. This approach opens new vistas for optoelectronic applications of MoWS_2_ nanomaterials in the mid-infrared waveband.

## Figures and Tables

**Table 1:** The key parameters for the saturable absorption characteristics of 2D MoWS_2_ SA.

Parameters	Values	Parameters	Values
*σ_g_*	6.98 × 10^−19^ cm^2^	*l_sa_*	10 nm
*σ_e_*	3.4 × 10^−19^ cm^2^	*n_s_*_0_	2.968 × 10^23^ cm^−3^
*τ_s_*	714.1 µs		

**Table 2:** Other parameters for theoretical solutions.

Parameters	Values	Parameters	Values	Parameters	Values
*σ*	2.5 × 10^−18^ cm^2^	*α*	3.8 cm^−1^	*t_ao_*	14 ns
*l*	10 mm	*λ_p_*	808 nm	*δ_a_*	1
*l_a_*	47 mm	*λ_s_*	1535 nm	*d_eff_*	−2.99 pm/V
*L_c_*	1195 mm	*λ_f_*	1064 nm	*h*	6.63 × 10^−34^ J·s
*l_opo_*	38 mm	*λ_id_*	3467 nm	$\varepsilon_{0}$	8.85 × 10^−12^ F/m
*l_KTA_*	20 mm	*n*	1.96	*K_c_*	5.1 W/mK
*w_p_*	200 μm	*n_a_*	1.53	*c*	3.0 × 10^8^ m/s
*w_l_*	187 μm	*n_s_*	1.385	*R_s_*	0.007
*w_g_*	184 μm	*n_f_*	1.367	*R_id_*	0.91
*w_a_*	206 μm	*n_i_*	1.4	*L*	0.1
*w_s_*	216 μm	*τ*	90 μs	*L_s_*	0.05
*w_k_*	141 μm	*τ_p_*	450 ps	*L_id_*	0.01
*w_id_*	141 μm	*f_a_*	0.43		

**Table 3:** A performance comparison of 2D nanomaterial-based intracavity OPOs driven by dual-loss-modulated QML lasers.

SA	Gain Medium	Nonlinear Crystal	Resonant Wave	Output Power	Pulse Width	Repetition Rate	Pulse Energy	Peak Power	Refs.
SWCNT	YVO_4_/Nd:YVO_4_	KTP	Signal wave	Signal wave: 373 mW	Signal wave: 119 ps	2 kHz	Signal wave: 124 μJ	Signal wave: 1.04 MW	[35]
Sb_2_Te_3_	YVO_4_/Nd:YVO_4_	KTA	Idler wave	Signal wave: 353 mW; Idler wave: 173 mW	Signal wave: 545 ps; Idler wave: 936 ps	1 kHz	Signal wave: 353 μJ; Idler wave: 173 μJ	Signal wave: 648 kW; Idler wave: 185 kW	[36]
MoWS_2_	YVO_4_/Nd:YVO_4_	KTA	Idler wave	Signal wave: 318 mW; Idler wave: 169 mW	Signal wave: 720 ps; Idler wave: 830 ps	1 kHz	Signal wave: 318 μJ; Idler wave: 169 μJ	Signal wave: 441.6 kW; Idler wave: 203.6 kW	This work

## Data Availability

The data presented in this study are available in this article.
